# Supplementary material for: Butyrate Conversion by Sulfate-Reducing and Methanogenic Communities from Anoxic Sediments of Aarhus Bay, Denmark
Source: Microorganisms. 2020 Apr 22;8(4):606. doi: 10.3390/microorganisms8040606 (PMC7232339; doi:10.3390/microorganisms8040606)
Supplement: Supplementary file 1 [file microorganisms-08-00606-s001.pdf]

Supplemental Information:

Table S1. Overview of reactions examined in this study. ΔG values were obtained from Thauer et al., 1977.

Reaction	Equation			ΔG°' (kJ/reaction)*
	**Acetogenic reactions**			
1	$Butyrate^{-} + 2\ H_2O$	→	$2\ Acetate^{-} + H^{+} + 2\ H_2$	+48.3
	**Sulfate-reducing reactions**			
2	$Butyrate^{-} + 0.5\ SO_4^{2-}$	→	$2\ Acetate^{-} + 0.5\ HS^{-} + 0.5\ H^{+}$	–27.8
3	$4\ H_2 + SO_4^{2-} + H^{+}$	→	$HS^{-} + 4\ H_2O$	–151.9
4	$Acetate^{-} + SO_4^{2-}$	→	$2\ HCO_3^{-} + HS^{-}$	–47.6
	**Methanogenic reactions**			
5	$4\ H_2 + HCO_3^{-} + H^{+}$	→	$CH_4 + 3\ H_2O$	–135.6
6	$Acetate^{-} + H_2O$	→	$CH_4 + HCO_3^{-}$	–31.0
	**Syntrophic butyrate conversion**			
7	$Butyrate^{-} + 0.5\ HCO_3^{-} + 0.5\ H_2O$	→	$2\ Acetate^{-} + 0.5\ CH_4 + 0.5\ H^{+}$	–19.5

Table S2. Overview of all the enrichment slurries fed with butyrate and the total amounts of the reactants consumed and products formed during the enrichment period. The enrichment slurries consisted of sediment either from sulfate zone (SZ), sulfate-methane transition zone (SMTZ) or methane zone (MZ) and were incubated at 25°C or 10°C, with 3 mM, 20 mM or without (-) sulfate amendments along the study. Slurries with * were presented in the butyrate conversion graphs and used for molecular analysis.

Sediment zone	Slurry Code	Treatment	Incubation temperature (°C)	Reactants (µmol/slurry)		Products (µmol/slurry)		
				Butyrate	Sulfate	Acetate	Sulfide	Methane
SZ	*B1	-	25	35085	313	37497	717	63347
	B2	-	25	38415	312	29386	508	64830
	B3	20 mM SO_4^{2-}	25	36896	37342	109911	39836	1030
	*B4	20 mM SO_4^{2-}	25	36816	35382	74468	33996	3252
	*B5	-	10	22092	351	24613	460	19853
	B6	-	10	24609	319	31487	364	12978
	*B7	20 mM SO_4^{2-}	10	19587	15551	33563	17064	93
	B8	20 mM SO_4^{2-}	10	22414	15282	35718	18012	0
SMTZ	*B1	3 mM SO_4^{2-}	25	33451	5500	10151	7479	73569
	B2	3 mM SO_4^{2-}	25	33748	8020	30128	9107	69486
	*B3	20 mM SO_4^{2-}	25	40754	45716	45255	38991	4183
	B4	20 mM SO_4^{2-}	25	41405	47734	46877	42512	3727
	B5	3 mM SO_4^{2-}	10	26272	6463	35148	7321	32045
	*B6	3 mM SO_4^{2-}	10	28158	6531	37574	7268	32483
	*B7	20 mM SO_4^{2-}	10	29226	35141	31129	36984	35
	B8	20 mM SO_4^{2-}	10	33078	31697	41505	32413	1015
MZ	B1	20 mM SO_4^{2-}	10	26059	18632	35892	14784	2956
	*B2	20 mM SO_4^{2-}	10	29488	34963	33448	38500	0
	*B3	20 mM SO_4^{2-}	25	54945	41587	91742	30618	5792
	B4	20 mM SO_4^{2-}	25	56743	54643	84195	39675	587
	*B5	-	25	45588	358	43022	261	105926
	B6	-	25	45829	782	29932	487	97903
	B7	-	10	18145	610	12727	380	27090
	*B8	-	10	16225	601	29455	424	8417

Table S3. The number of reads per sample generated by Pyrosequencing for *Bacteria* and HiSeq Illumina sequencing for *Archaea*. ENV: Original Sediment Sample

Origin	Slurry	Bacterial reads	Archaeal reads
Sulfate zone	ENV	8787	9120
	B4	12393	88555
	B1	18687	121246
	B7	12007	61722
	B5	12194	89283
Sulfate-methane transition zone	ENV	3202	18696
	B1	3758	75708
	B3	5167	47520
	B6	5443	42599
	B7	7198	58464
Methane zone	ENV	10903	30848
	B3	15094	39087
	B5	11718	136909
	B2	11686	52551
	B8	10891	94124

Table S4. Relative abundance (HPLC/MS mass chromatogram area, summed by IPL head group per gram sediment dry weight) of all IPLs identified in Aarhus Bay sediment and the enrichment slurries inoculated with this sediment. SZ: Sulfate zone; SMTZ: Sulfate Methane Transition Zone; MZ: Methane zone; Sediment depth: 120 cm = sulfate zone, 135-165 cm = sulfate-methane transition zone, 180-225 cm = methane zone.

Origin	Sample	Mhex	PA	DH	Betaine	DMPE	PE	PG	OL	GlcA	PC	TMO	PI	LPC
SZ	**B1**	0	0	0	0	0	1946176	0	0	0	0	0	0	2954044
	**B4**	0	0	7594944	0	0	1983971	0	0	0	2832247	0	0	849552
	**B5**	0	0	11499437	0	0	4455602	0	0	0	0	0	0	1204249
	**B7**	0	0	0	0	0	10303739	17291589	0	0	1499265	0	0	0
SMTZ	**B1**	39551686	0	12878869	0	0	12799850	1060523	0	35097613	8812082	0	0	590925
	**B3**	0	0	5197585	0	0	5120743	11400411	1174897	0	0	0	0	0
	**B6**	0	0	12235720	0	0	10403673	47667216	0	0	31353022	0	0	711956
	**B7**	0	0	0	0	0	1494608	24104131	0	0	0	0	0	0
MZ	**B1**	0	0	485170	0	0	17021650	18855340	0	0	757859168	15574565	0	16962049
	**B2**	0	68529	0	0	0	1153218	8106805	0	0	0	0	467142	0
	**B3**	0	0	1648930	0	0	2230226	8895759	0	0	0	0	0	1088251
	**B4**	0	0	0	0	0	0	3354888	0	0	110589668	17064261	0	23535905
	**B5**	737203	0	21381687	11195317	2801118	0	0	0	0	12704704	1724058	0	2048403
	**B6**	0	613445	16440021	0	0	4403271	0	0	0	0	0	0	0
	**B7**	0	0	0	0	0	0	0	0	0	0	0	0	0
	**B8**	0	365393	6954652	0	0	1906084	0	0	0	0	0	139512	401081
Sediment depth	**120 cm**	0	531101	0	0	0	1207772	0	0	0	0	0	1131887	0
	**135 cm**	0	443604	0	0	0	0	0	0	0	0	0	0	0
	**150 cm**	0	552721	0	0	0	808333	0	0	0	0	0	0	0
	**165 cm**	0	738732	0	0	0	0	0	0	0	0	0	0	0
	**180 cm**	0	1255773	0	0	0	0	0	0	0	0	0	0	0
	**195 cm**	0	373889	0	0	0	0	0	0	0	0	0	0	0
	**210 cm**	0	473870	0	0	0	2551160	0	0	0	0	0	0	0
	**225 cm**	0	247824	0	0	0	0	0	0	0	0	0	0	0

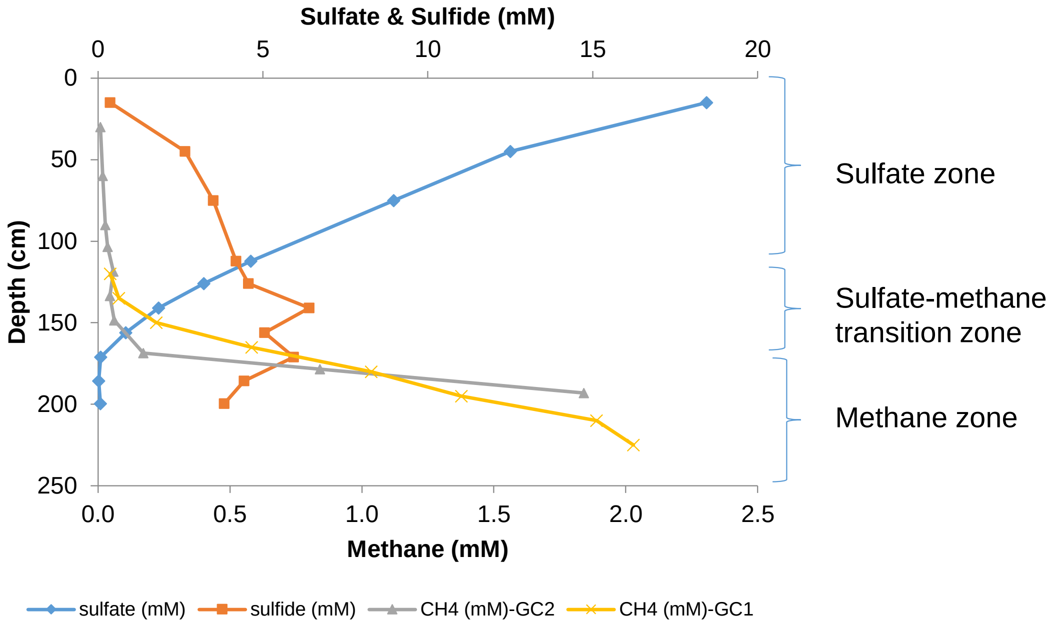

Figure S1. Depth profiles of sediment pore water sulfate, sulfide and methane for Station M1, in Aarhus Bay, Denmark. Methane-GC1 and Methane-GC2 stands for methane concentrations retrieved from two different gravity corers, gravity corer 1 and 2, respectively.

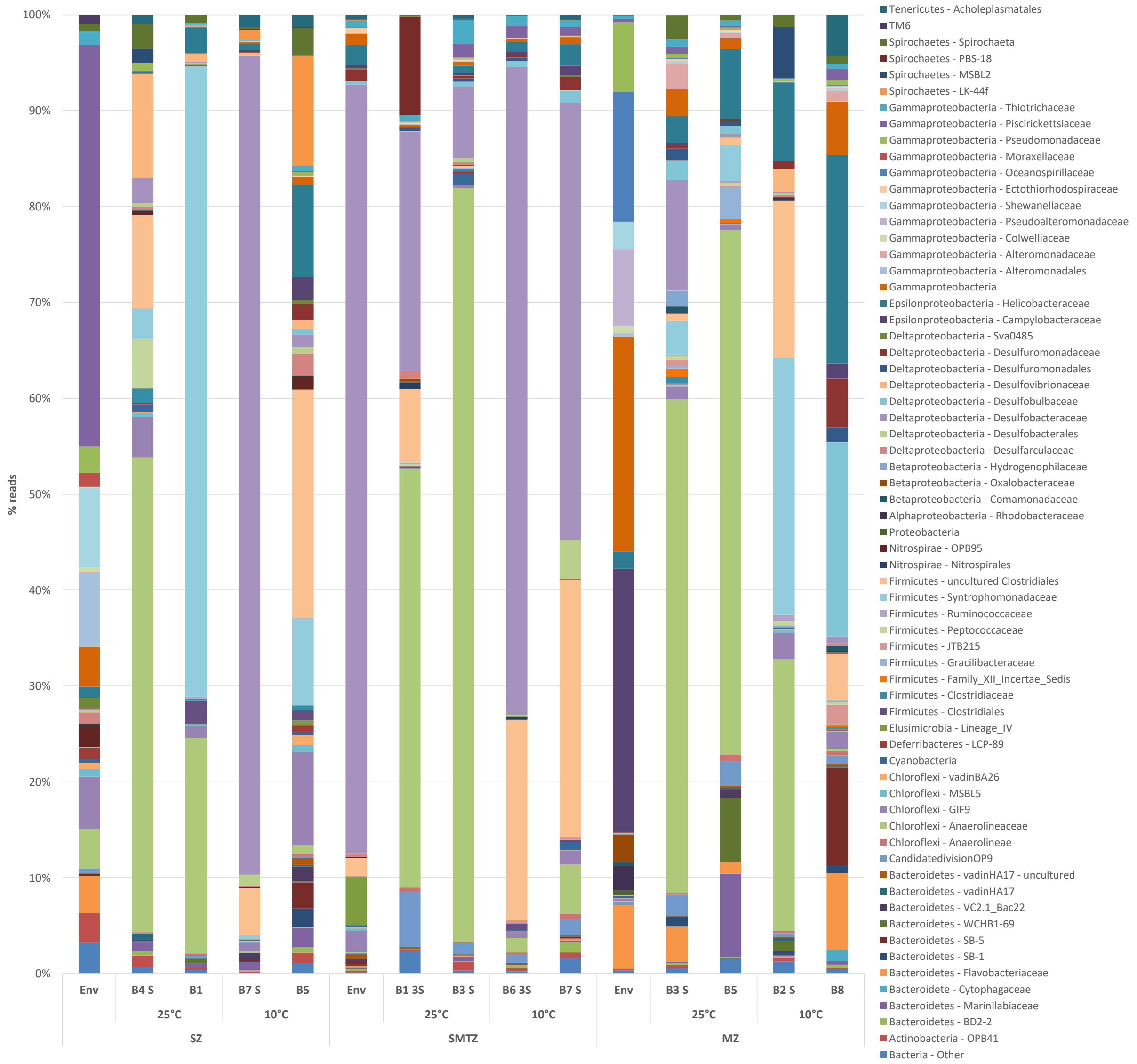

Figure S2. Relative abundance of the bacteria in all slurries and environmental samples at family level, normalized to 100%. Only those families that were present at an abundance >1% in at least one sample were included. SZ: Sulfate zone, SMTZ: Sulfate-methane transition zone; MZ: Methane zone, Env: Original Sediment Sample belonging to the indicated biogeochemical zone. S: 20mM sulfate, 3S:3mM sulfate is used as electron acceptor in slurries. Slurries that were not labeled with 'S' or '3S' were incubated without sulfate.

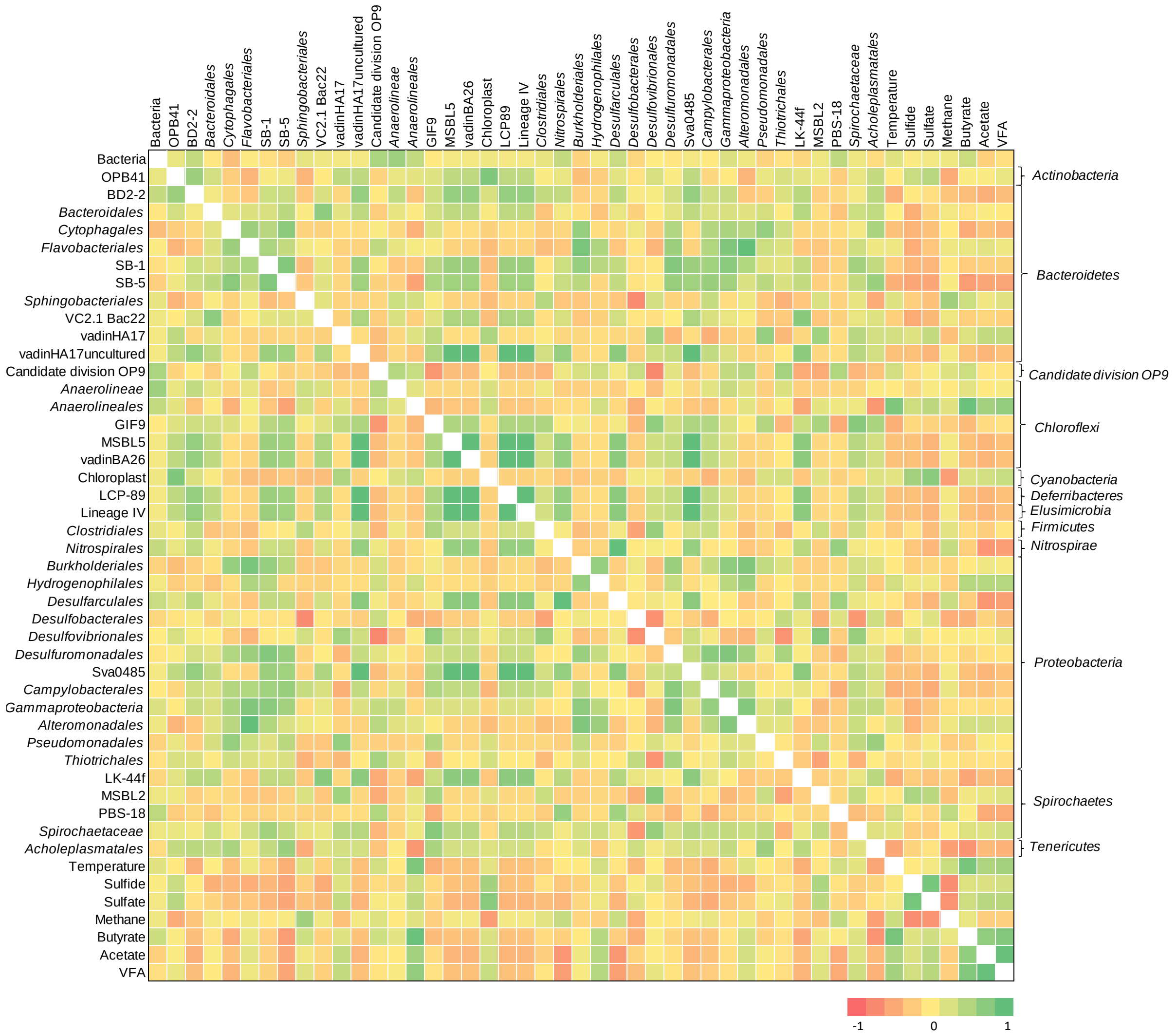

Figure S3. Heatmap representing the correlation between bacterial orders present at a relative abundance >1% of total reads across the 12 slurry samples analyzed and experimental parameters. Correlations were determined by means of the two tailed Spearman's Rank Order Correlation test. The heatmap colors represent the relative percentage of the microbial order assignments. Square colors shifting towards bright green indicate strong correlation.

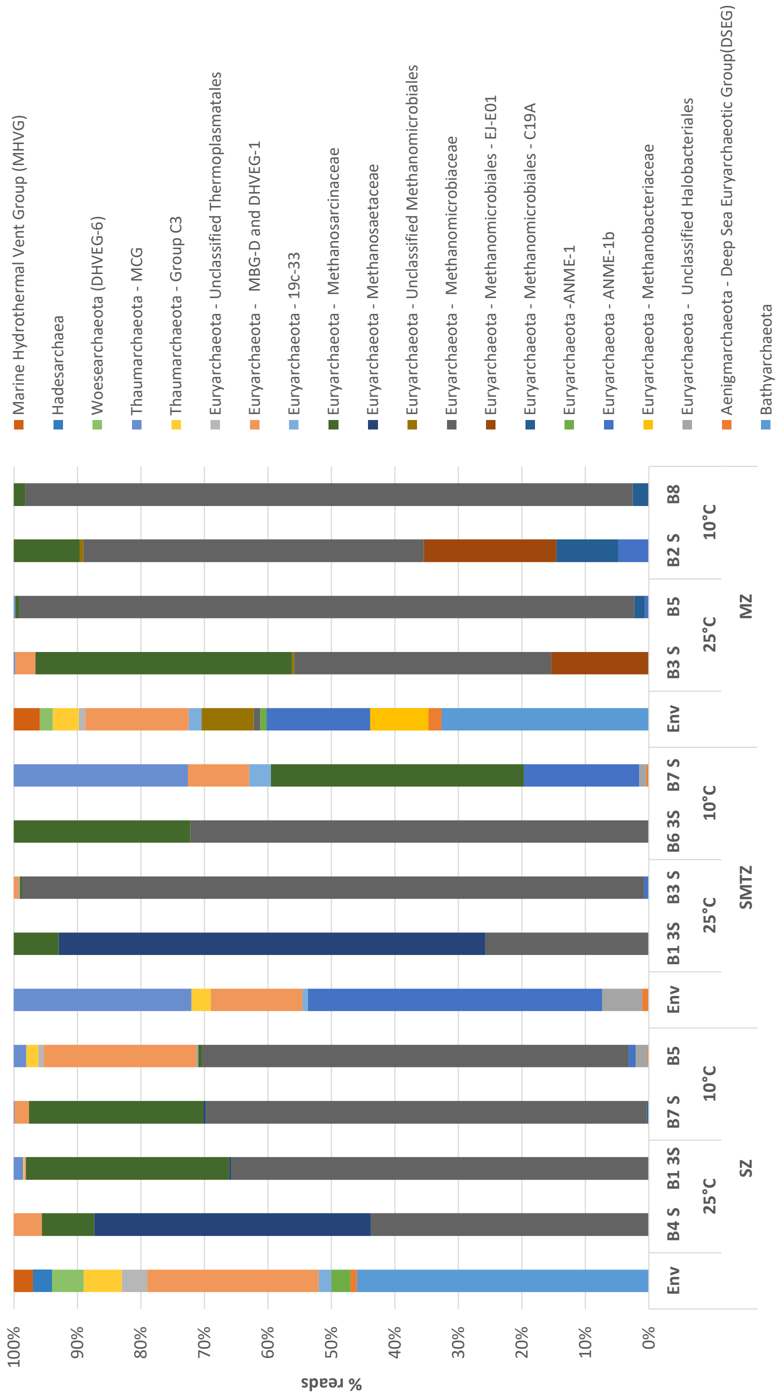

Figure S4. Relative abundance of the *Archaea* in all slurries and environmental samples at family level, normalized to 100%. Only those families that were present at an abundance >1% in at least one sample were included in the graph. SZ: Sulfate zone, SMTZ: Sulfate-methane transition zone; MZ: Methane zone. Env: Original Sediment Sample belonging to the indicated biogeochemical zone. S: 20mM sulfate, 3S:3mM sulfate is used as electron acceptor in slurries. Slurries that were not labeled with 'S' or '3S' were incubated without sulfate.

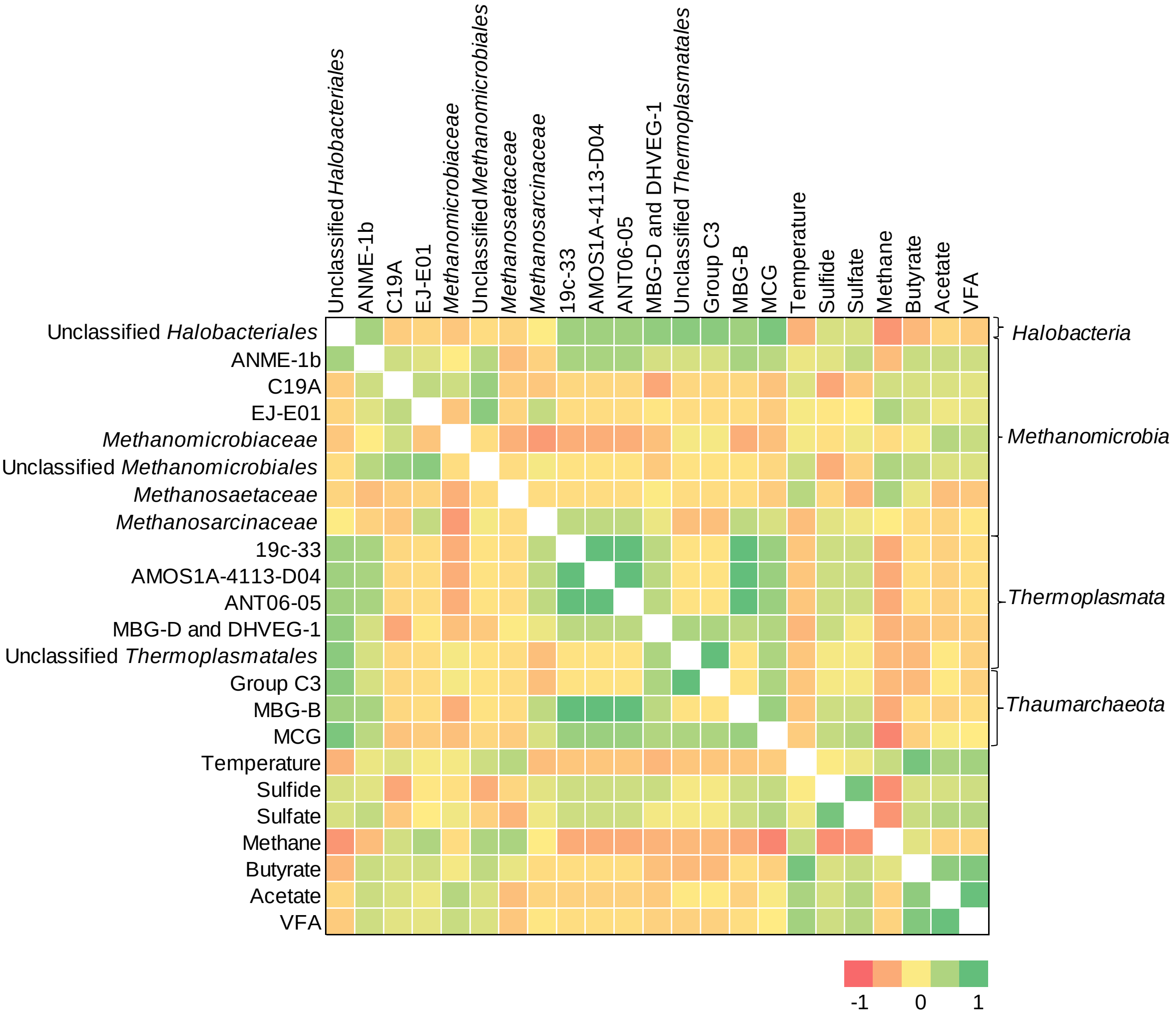

Figure S5. Heatmap showing the correlation between archaeal families present at a relative abundance >1% of total reads across the 12 slurry samples analyzed and experimental parameters. Correlations were determined by means of the two tailed Spearman's Rank Order Correlation test. The heatmap colors represent the relative percentage of the microbial family assignments. Square colors shifted towards bright green indicate strong correlation.

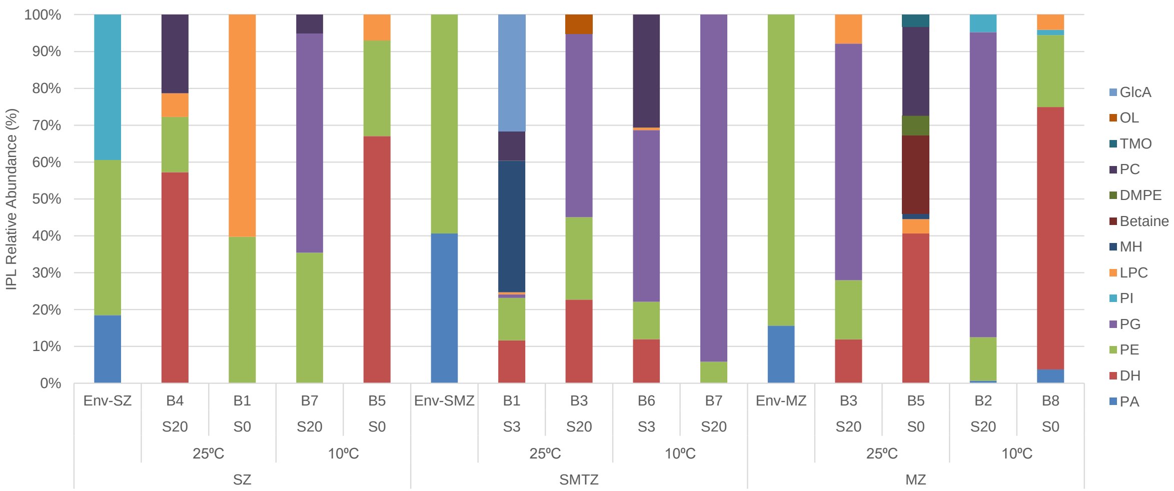

Figure S6: Percent relative abundance of IPLs in all enrichment slurries and environmental samples used for molecular analysis. Env = Original Sediment Samples belonging to the indicated biogeochemical zone, S20 = 20 mM sulfate added, S0 = 0 mM sulfate added, S3 = 3 mM sulfate added, °C indicates incubation temperature, SZ = sulfate zone, SMTZ = sulfate-methane transition zone, MZ = methane zone.

IPL key: PA = phosphatidic acid, DH = dihexose, PE = phosphatidylethanolamine, PG = phosphatidylglycerol, PI = phosphatidylinositol, LPC = lyso-phosphatidylcholine, MH = monohexose, DMPE = dimethylphosphatidylethanolamine, PC = phosphatidylcholine, TMO = trimethylornithine, OL = ornithine, GlcA = glucuronic acid.

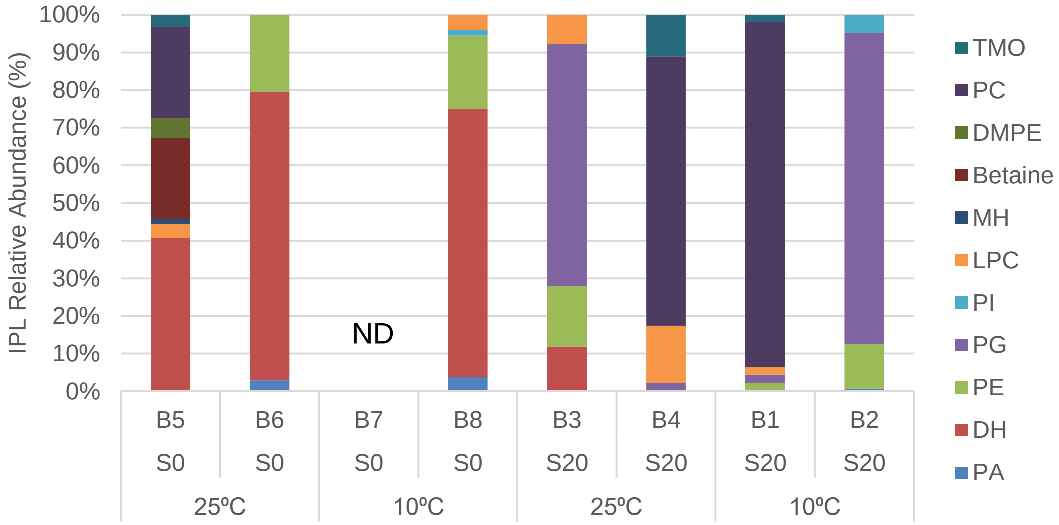

Figure S7: Percent relative abundance of IPLs in all **methane zone enrichment slurries**. IPL relative abundances for all methane zone slurries are combined to show full comparison between slurries. S20 = 20 mM sulfate added, S0 = 0 mM sulfate added, °C indicates incubation temperature, ND: Not Determined.

IPL key: PA = phosphatidic acid, DH = dihexose, PE = phosphatidylethanolamine, PG = phosphatidylglycerol, PI = phosphatidylinositol, LPC = lyso-phosphatidylcholine, MH = monohexose, DMPE = dimethylphosphatidylethanolamine, PC = phosphatidylcholine, TMO = trimethylornithine.

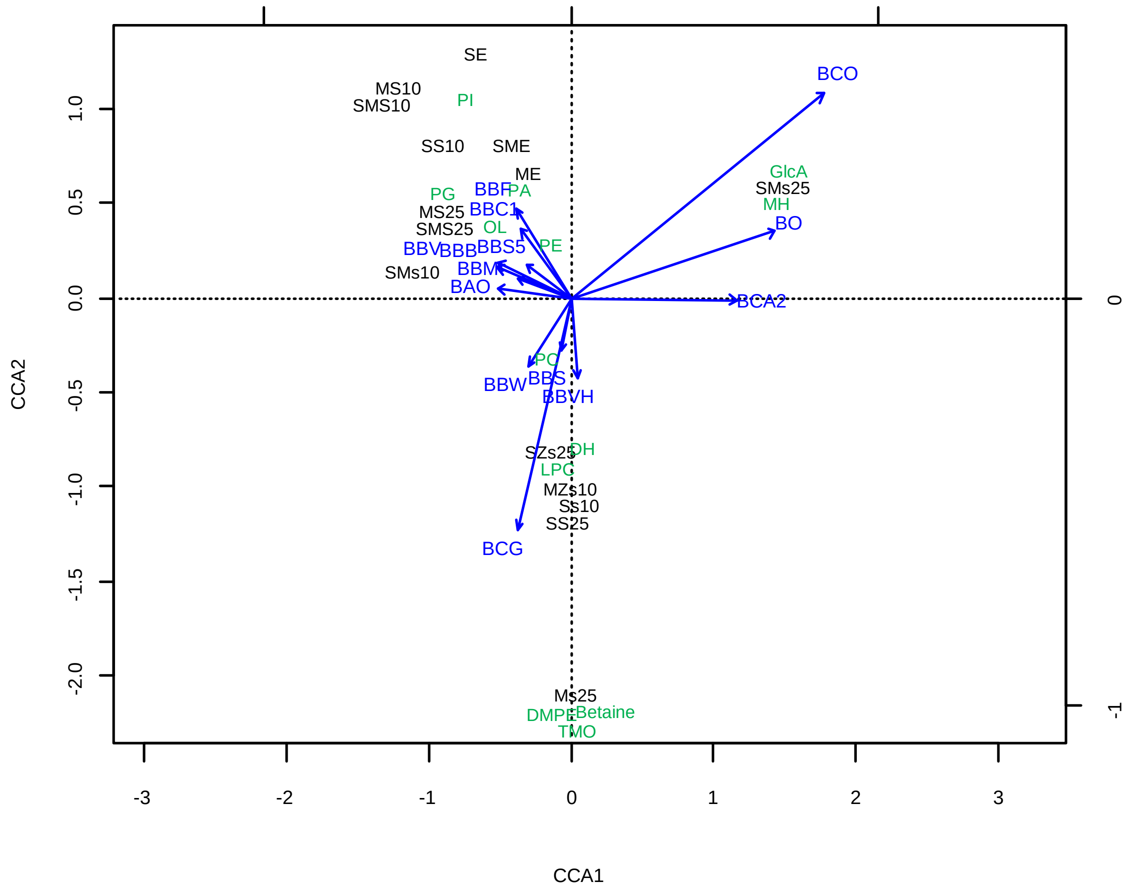

Figure S8: Canonical correspondence analysis of intact polar lipid (IPL) distribution, bacterial taxa distribution, and growth conditions for all enrichment slurries.

Black text, culture condition key: S = sulfate zone, SM = sulfate-methane transition zone, M = methane zone, S = high sulfate amendment, s = low sulfate amendment, 25 = 25°C, 10 = 10°C.

Green text, IPL key: MH = monohexose, PA = phosphatidic acid, DH = dihexose, DMPE = dimethylphosphatidylethanolamine, PE = phosphatidylethanolamine, PG = phosphatidylglycerol, OL = ornithine, GlcA =glucuronic acid, PC = phosphatidylcholine, TMO = trimethylornithine, MMPE = monomethylphosphatidylethanolamine, PI = phosphatidylinositol, LPC = lyso-phosphatidylcholine.

Blue text, bacterial taxa key: BAO = Bacteria - *Actinobacteria* - OPB41; BBB = Bacteria - *Bacteroidetes* - BD2-2; BBC1 = Bacteria - *Bacteroidetes* - *Cytophagaceae*; BBF = Bacteria - *Bacteroidetes* - *Flavobacteriaceae*; BBM = Bacteria - *Bacteroidetes* - *Marinilabiaceae*; BBS =

Bacteria - *Bacteroidetes* - SB-1; BBS5 = Bacteria - *Bacteroidetes* - SB-5; BBV = Bacteria - *Bacteroidetes* - VC2.1_Bac22; BBVH = Bacteria - *Bacteroidetes* - vadinHA17; BBW = Bacteria - *Bacteroidetes* - WCHB1-69; BCA2 = Bacteria - *Chloroflexi* - *Anaerolineaceae*; BCG = Bacteria - *Chloroflexi* - GIF9; BCO = Bacteria - Candidate division OP9; BO = Bacteria other.
